# Strengthening Social and Behavior Change Programming Through Application of an Adaptive Management Framework: A Case Study in Tanzania

**DOI:** 10.9745/GHSP-D-22-00215

**Published:** 2023-12-18

**Authors:** Claire Gillum, Kara Tureski, Joseph Msofe

**Affiliations:** a FHI 360, Washington, DC, USA.; b FHI 360, Dar es Salaam, Tanzania.

## Abstract

Integrated social and behavior change (SBC) programs seek to achieve changes in behaviors and norms across a range of health areas. Realizing changes across multiple behaviors is inherently complex and requires programs to be designed and implemented nimbly in ways that are responsive to feedback, new learning, and contextual shifts. Responsive feedback (RF) promotes just this kind of data analysis, use, and programmatic adaptation. Under the U.S. Agency for International Development (USAID)'s flagship SBC program in Tanzania, USAID Tulonge Afya, we applied FHI 360's SBC Adaptive Management Framework, which shares many similarities with RF. It includes meaningful engagement with an array of stakeholders throughout project design, implementation, monitoring, and evaluation (M&E); requires close collaboration between M&E and programmatic staff to ensure data collection and use are informed by and contribute to refinement of the project's theory of change; and incorporates purposeful, routine opportunities for learning and course correction. Accompanying the framework is a suite of tools and activities to facilitate systematic collection, analysis, and application of data for project design, improvement, and evaluation. Use of the framework within a complex, integrated SBC program improved our ability to address emerging priorities and achieve greater scale, saturation, and quality execution of activities. It also facilitated significant input from local communities and stakeholders—through shared data analysis and feedback loops—ensuring project activities reflected their strengths, needs, and preferences rather than the perspectives and biases of program implementers. In this article, we discuss the key components of the SBC Adaptive Management Framework and its commonalities with RF, how use of the framework and the underlying principles and elements of the RF approach contributed to the project's success, and share lessons that may strengthen and expand the application of RF and similar approaches within future SBC programs.

## Abstract (precis)

The SBC Adaptive Management Framework, tested under the USAID Tulonge Afya project in Tanzania, offers a systematic, evidence-informed, and participatory approach to strengthen program design and implementation and enhance outcomes.

## Teaser

**Key Findings:**

- Sharing many features with the responsive feedback approach, the social and behavior change (SBC) Adaptive Management Framework was developed to guide implementers in applying a systematic approach for the use of data to design, monitor and improve, and evaluate program activities.
- Within a complex, integrated SBC project, U.S. Agency for International Development Tulonge Afya, the SBC Adaptive Management Framework was successfully applied, contributing to key improvements in the scale, saturation, and quality of activities.

**Key Implications:**

- For programs to effectively apply an adaptive management approach, both donors and program implementers should ensure sufficient allocation of time and resources to these activities throughout the project life cycle.
- Creating a culture of testing and learning requires program implementers to be open to diverse perspectives and have their assumptions challenged, recognizing that this has tangible benefits for project delivery and broader learning.

## INTRODUCTION

The field of social and behavior change (SBC) uses evidence-based strategies to address individual, interpersonal, social, and/or structural determinants of behaviors, applying mutually reinforcing approaches to achieve changes in behaviors or social conditions. SBC strategies are applied within health and development programs globally to drive demand for health services, increase sustained uptake of home health and preventative behaviors, and improve enabling environments for responsive, equitable, and transformative programs and services. SBC uses a range of strategies, including behavior change communication, social mobilization, advocacy, quality improvement (QI), and other approaches grounded within the social and behavioral sciences.

Over the past decade, appreciation has increased for the intersection of health with other sectors and recognition of the need for improved strategies to address development complexities using multifaceted interventions. The field of SBC, which addresses the complex interplay between factors that influence behaviors across levels, is no exception. Large-scale integrated SBC programs seek to address barriers and leverage facilitators to achieve a range of behavior change objectives focused on health and development. They frequently combine the promotion of healthy behaviors across 2 or more areas and increasingly seek to achieve an even greater number of SBC objectives, both within and among sectors. Such programs require complex, multilevel strategies. Measurement of change—including the adoption or maintenance of particular behaviors and the social conditions that inhibit or facilitate them—is challenging. Also challenging is the identification of factors for program successes or gaps. These may range from challenges with implementation—such as fidelity of implementation to program design or the quality of implementation—to overall SBC strategy design issues.

In response to these challenges, FHI 360 developed the SBC Adaptive Management Framework, which provides a roadmap and tools to facilitate the routine use of near real-time data alongside participatory analysis approaches for project design and evaluation, as well as to understand project gaps and achievements and identify and implement project adaptations in response.

The SBC Adaptive Management Framework provides a roadmap and tools to facilitate routine use of near real-time data alongside participatory analysis approaches for project design and evaluation.

Although the framework was developed independent of the responsive feedback (RF) approach, they share many similarities (Table 1). They are both centered around a systematic process for data capture, analysis, and program adaptation, with a strong emphasis on generating learning that will contribute to overall project improvements. Both approaches emphasize the importance of engaging a diverse array of stakeholders—including funders, program designers, implementers, and monitoring, evaluation, research, and learning (MERL) teams—in learning and adaptation. Each comprises a set of core tools and approaches, such as pause and reflect sessions, that aim to facilitate purposeful and timely data and evidence collection, analysis, and application. A theory of change is also central to both RF and the framework. However, we note that a theory of change is developed as part of the RF approach, whereas the SBC Adaptive Management Framework is applied to and refines a project's preexisting theory of change. There are other subtle distinctions between the framework and RF. For example, unlike RF, the framework does not formalize stakeholder roles and responsibilities in a documented plan nor does it incorporate an explicit step to establish and prioritize learning questions. The framework emphasizes data collection at each step in its process, incorporating traditional baseline, midline, and endline survey methodologies with more nimble and routine data and evidence collection and use. The framework also extends beyond identifying adaptive actions to be taken—the final step in the RF process—and incorporates steps related to documenting adaptive actions, ensuring they are implemented with quality, and evaluating project results.

**TABLE 1.** Similarities Between the Responsive Feedback Approach^1^^,^^2^ and the SBC Adaptive Management Framework

	Responsive Feedback Approach	SBC Adaptive Management Framework
Process	A streamlined, systematic 5-step process that guides stakeholder engagement, data collection and review, and action planning	An iterative 5-step cycle that guides data collection, use, and program adaptation throughout a project life cycle
Stakeholder engagement	Integrates purposeful, inclusive, and non-siloed engagement of all stakeholders in learning and adaptation	Integrates purposeful, inclusive, and non-siloed engagement of all stakeholders in learning and adaptation
Theory of change	Rooted in a theory of change that continuously questions and tests assumptions on which interventions are based	Aligns data collection and use with a project's theory of change to focus and prioritize learning for improved SBC impact
Core tools	Stakeholder engagement plan Theory of change Learning questions and research plan Pause and reflect sessions Lessons and action plans	Data collection tools (qualitative and quantitative) Dashboards and data visualizations Decision-making tools Adaptive management trackers Quality assurance standards

Abbreviation: SBC, social and behavior change.

Recognizing these similarities and differences between RF and the SBC Adaptive Management Framework, we present this case study as an example of how the underlying principles and elements of the RF approach contributed to success within a large, complex integrated health SBC program and share lessons that may strengthen and expand the application of RF and similar approaches within future SBC programs.

## USAID TULONGE AFYA PROJECT DESCRIPTION

Implemented by FHI 360 from 2017 to 2022, the USAID Tulonge Afya project—Kiswahili for “Let's Talk about Health”—was the USAID flagship SBC project in Tanzania, delivering integrated activities that addressed SBC needs across 6 core health areas: HIV; family planning and reproductive health; malaria; maternal, newborn, and child health; TB; and emerging infectious diseases. The project's goal was to catalyze opportunities for Tanzanians to improve their health status by transforming sociocultural norms and supporting the adoption of healthier behaviors. Through use of a participatory, evidence-based, and theory-informed approach, the project developed 2 integrated, branded SBC platforms: Naweza (Kiswahili for “I Can”) targeted adults at key life stages (pregnancy and caregiving for a child aged younger than 5 years, with a focus on the first 1,000 days); and Sitetereki (“Unshakeable”) engaged youth to increase uptake of positive sexual and reproductive health behaviors. The Box includes examples of the behaviors promoted under the Naweza Pregnancy Life Stage Package. These integrated platforms were supplemented by a long-running HIV-focused campaign, Furaha Yangu (“My Happiness”), which addressed the needs of priority populations at higher risk of HIV and people living with HIV. From 2019, the project implemented comprehensive packages of SBC activities under these platforms, including mass and social media at the national level; mid-media (e.g., community theater, radio, and events); community mobilization; and interpersonal communication activities (e.g., community health worker counseling and small group dialogues) in collaboration with local civil society organization (CSO) partners in 29 focal districts.

The USAID Tulonge Afya project sought to transform sociocultural norms and support the adoption of healthier behaviors among Tanzanians.

BOX Behaviors Promoted Under the Naweza Social and Behavior Change Platform Pregnancy Life Stage Package

- Go early, attend, and complete more than 4 antenatal care visits (8 contacts are desired)
- Take 3 doses of intermittent preventative therapy during antenatal care visits
- Sleep under an insecticide-treated net every night
- If HIV positive, attend prevention of mother-to-child transmission services and take antiretroviral therapy
- Attend a health facility for delivery
- Attend postnatal care visits and seek prompt and appropriate care at the health facility upon the first sight of postpartum danger signs
- Initiate breastfeeding within the first hour of birth
- Talk with your health care provider about postpartum family planning options
- Bring your infant to the facility for an early visit at 4–6 weeks and for HIV testing if mother is positive or status unknown

## SBC ADAPTIVE MANAGEMENT FRAMEWORK

### Framework Overview

Attribution of behavior or social change results to SBC programs or specific SBC interventions is difficult.^3^ Challenges include the multitude of factors that impact social and behavioral outcomes; the presence of numerous actors and programs working in the same geographic area on the same or related issues; and the limitations of many existing behavioral measures, which often rely on audience recall and self-report. This complexity often leads large, integrated SBC programs to emphasize the collection and routine use of process and output indicators rather than intermediary and outcome-related indicators, with the exception of traditional SBC surveys at baseline, endline, and occasionally midline.

**FIGURE 1:**
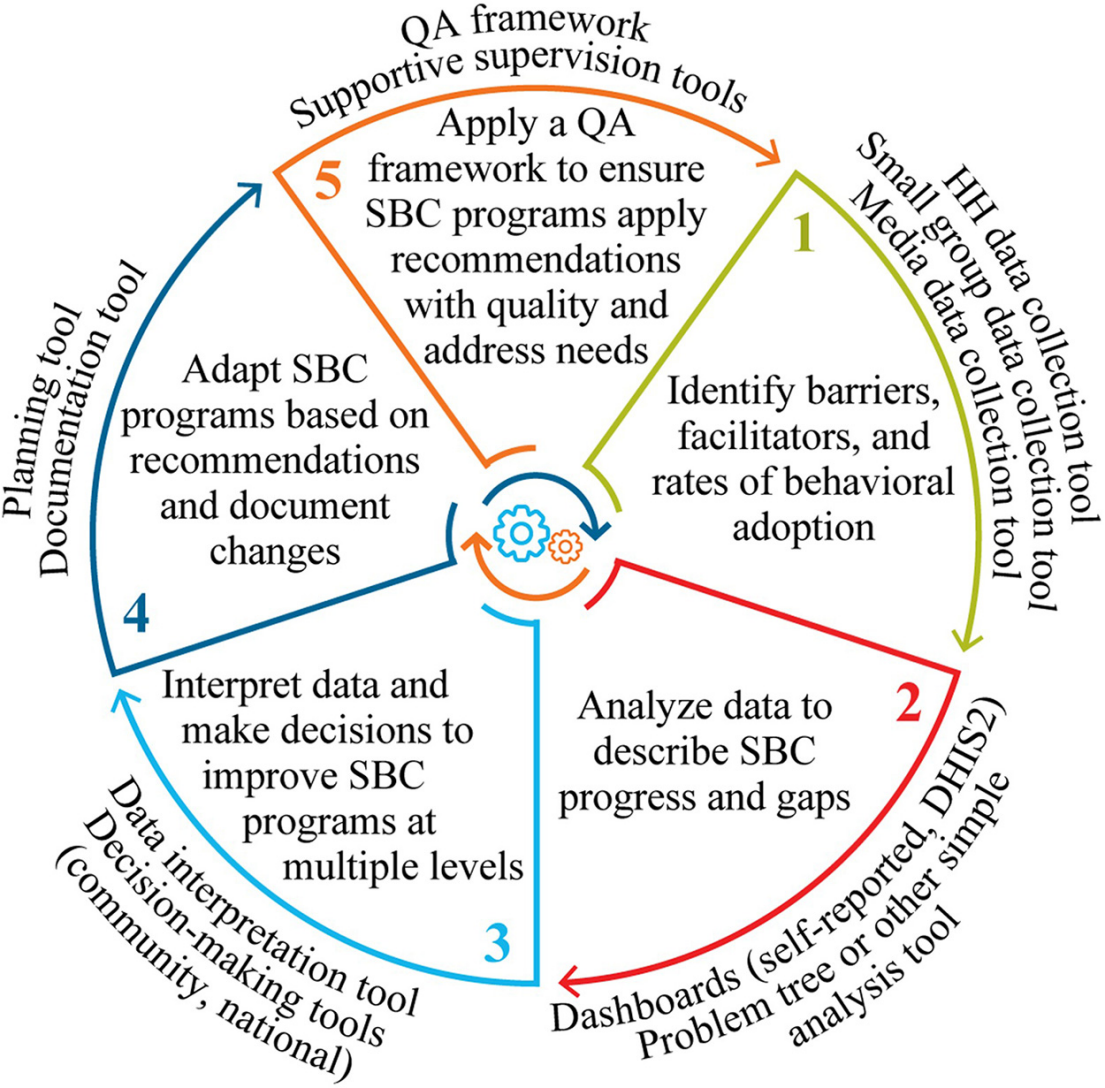
SBC Adaptive Management Framework Abbreviation: HH, household; QA, quality assurance; SBC, social and behavior change.

In response, FHI 360 developed the SBC Adaptive Management Framework (Figure 1) specifically to: (1) inform strategy design and evaluation, including understanding and elucidating pathways to behavior change; (2) identify SBC program successes and challenges against performance indicators, including intermediary and outcome-level indicators; (3) measure performance and understand implementation fidelity and quality across program sites; (4) strengthen SBC programs to enable responsive shifts, as needed; and (5) adapt programs to meet contextual needs and/or replicate or scale up successful strategies. Implementation of the SBC Adaptive Management Framework embodies many of the principles of RF and enables a systematic approach to using data to design, monitor and improve, and evaluate program activities and describe impact.

#### Components of the Framework

Each step of the SBC Adaptive Management Framework describes when data should be collected and used to inform the adaptive management needs of SBC programs. As with RF, each step is designed to purposefully engage a wide array of stakeholders and all project staff to maximize inclusion in learning and adaptation activities (Figure 1).

1. Step 1: To support the first framework step, a variety of different data collection approaches may be used. These can include household-level surveys, omnibus and sentinel surveys, qualitative research, and codesign sessions with priority populations and stakeholders to understand needs and identify and codesign solutions for change. This step is conducted in collaboration with program and MERL teams, relevant host country government officials, local implementing partners, and priority populations or stakeholder groups.
2. Step 2: Data, including intermediary and outcome data, are collected routinely as part of SBC program implementation. For FHI 360 projects, including USAID Tulonge Afya, this includes regular collection of program M&E data, frequent behavioral surveys, ongoing analysis of service delivery and other health management information system data, and routine analysis of feedback from priority audiences derived from program feedback loops (e.g., comments and questions from radio show listener call-ins or generated in small group dialogue sessions). This aligns with the RF approach, which encourages use of data and feedback from a range of sources to answer learning questions.
3. Step 3: Subsequently, similar to the final stage in the RF approach, data are then reviewed by and with a diverse set of stakeholders to identify what is working and where there are opportunities for improvement against predefined indicators. This includes quarterly review of output-related indicators and changes in behavioral determinant-related indicators against targets and aligned with a project's theory of change.
4. Step 4: From this, review teams make recommendations for program changes and record them in an adaptive management change tracking tool.
5. Step 5: Implementation of adaptative actions is then monitored according to predefined SBC quality assurance (QA) standards, which set expectations and benchmarks for the design and delivery of SBC interventions.

As with RF, each step of the framework is designed to engage a wide array of stakeholders and all project staff to maximize inclusion in learning and adaptation activities.

While FHI 360 and the USAID Tulonge Afya project originally designed the SBC Adaptive Management Framework and tools to meet program needs, we engaged stakeholders as appropriate at various junctures in helping to advance and refine the stages described in the framework and the related tools.

### Application of the Framework Within USAID Tulonge Afya

Application of the framework under USAID Tulonge Afya began in the early stages of project development, guiding formative research plans and the establishment of the project's M&E system. We used the framework to consider and prioritize the indicators the project would track, identify the tools and systems we would need to gather and apply these data, and establish appropriate time points for carrying out pause and reflect activities at the district, zonal, and national levels. As the project moved into implementation of its SBC strategies, we regularly used the SBC Adaptive Management Framework—for example, during internal quarterly planning meetings, annual work planning, and strategy reviews (Table 2)—as a touchstone to determine whether we were collecting and using data, learning, and feedback effectively and according to project plans. By using the framework as a core project planning tool, we ensured that workplans and activity implementation plans incorporated adaptive management activities from the outset, rather than being considered only once challenges arose. When new activities were being designed and integrated within the program, the framework served as a prompt to ensure tools were updated to reflect any new MERL needs. Throughout the course of implementation, whenever the project received feedback on its strategies or activities, the framework provided tools to ensure this feedback was systematically recorded and accessible to decision-makers. By linking back to implementation (Step 5), the framework also provided QA tools to ensure that adaptive actions were integrated within project implementation with quality. During the final year of project implementation, the information captured through application of the framework enabled the project to examine the impacts of strategic changes and adaptive actions on the project's social and behavioral objectives. Had only output, midline, and endline data been available, the full understanding of program shifts and their impacts would not have been possible.

**TABLE 2.** Tools Applied by the USAID Tulonge Afya Project as Part of Its Adaptive Management Approach and Stakeholders Engaged

SBC Adaptive Management Framework Step	Tools	Stakeholders Engaged
1. Identify barriers, facilitators, and rates of behavioral adoption	Baseline/endline household survey tool Midline/endline qualitative feedback Routine (quarterly, annual) surveys to track priority indicators tied to the project's theory of change Frequently asked questions tracker	Community members Local CSO partners and volunteers GOT staff USAID/Tanzania and USAID/Washington USAID Tulonge Afya team (SBC, MERL, CS, management/operations; in-country and HQ teams)
2. Analyze data to describe SBC progress and gaps	Data dashboards with simple visualizations for priority indicators	Local CSO partners and volunteers GOT staff USAID/Tanzania USAID Tulonge Afya team (SBC, MERL, CS, management/operations in-country and HQ teams)
3. Interpret data and make decisions to improve SBC programs at multiple levels (e.g., implementation/process-related changes, strategy-related changes)	Monthly check-ins with community partners Quarterly data review meetings Routine pause and reflect sessions tied to annual work planning and strategy reviews	Local CSO partners and volunteers GOT staff USAID/Tanzania and USAID/Washington USAID Tulonge Afya team (SBC, MERL, CS, management/operations in-country and HQ teams)
4. Adapt SBC programs based on recommendations and document changes	Adaptive management tracker Codesign workshops for adaptive actions	Community members Local CSO partners and volunteers GOT staff USAID/Tanzania USAID Tulonge Afya team (SBC, MERL, CS, management/operations in-country and HQ teams)
5. Apply a quality assurance framework to ensure SBC programs apply recommendations with quality and address needs	Supportive supervision mobile application SBC quality assurance standards Routine pause and reflect sessions tied to annual work planning and strategy reviews	Community members Local CSO partners and volunteers GOT staff USAID/Tanzania and USAID/Washington USAID Tulonge Afya team (SBC, MERL, CS, management/operations in-country and HQ teams)

Abbreviations: CS, capacity-strengthening; CSO, civil society organization; GOT, Government of Tanzania; HQ, headquarters; MERL, monitoring, evaluation, research, and learning; SBC, social and behavior change; USAID, U.S. Agency for International Development.

Adaptive management activities were incorporated into workplans and activities from the outset, rather than being considered only once challenges arose.

Table 2 summarizes the tools USAID Tulonge Afya developed and used in its application of the SBC Adaptive Management Framework and the stakeholders engaged at each step. While many of these tools are common to other adaptive management and QI approaches, we found few examples of such tools developed or adapted for SBC programming. In many cases, existing available tools—such as dashboards, tracking tools, and supportive supervision checklists—are tailored for service delivery programs and QI initiatives. The tools listed were developed specifically to meet the needs and requirements of SBC programs, taking into consideration relevant SBC indicators and data sources.

**FIGURE 2:**
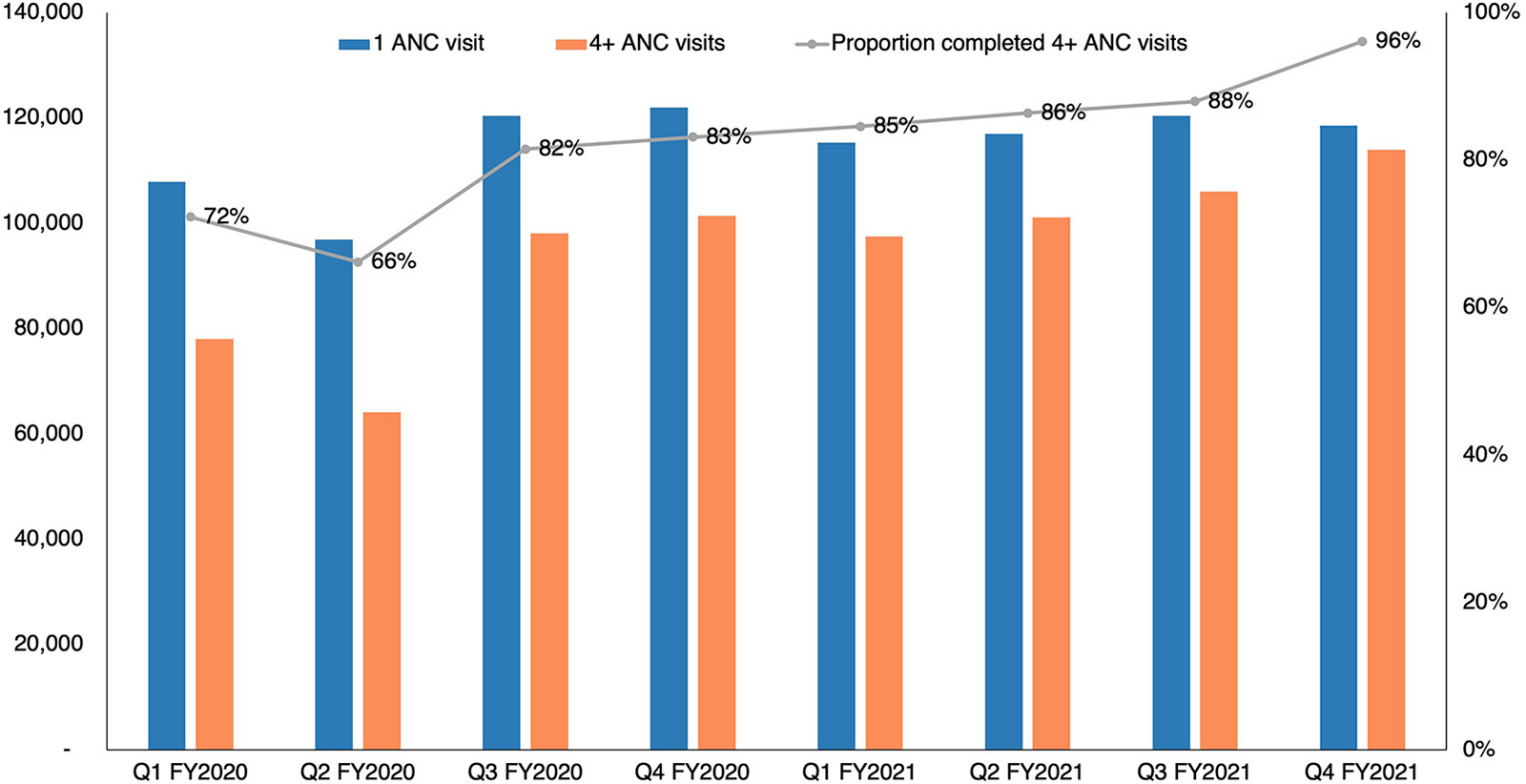
Completion of 4 or More ANC Visits in 29 USAID Tulonge Afya–Supported Districts^a^ Abbreviation: ANC, antenatal care; FY, fiscal year; Q, quarter; USAID, U.S. Agency for International Development. ^a^Source: DHIS2.

We recognized that relying on a single source of data was unlikely to help us understand project progress and impacts and that different stakeholders could contribute diverse and complementary perspectives on project implementation and necessary adaptations. In response, as outlined in Table 2, we went beyond typical stakeholder engagement approaches, such as national-level pause and reflect sessions, and instead used the framework to engage stakeholders more fully and routinely in providing feedback, reviewing data, and identifying adaptive actions to be taken. To maximize the effectiveness of this engagement, our tools and strategies sought to reflect that different stakeholder groups have varying levels of comfort with data interpretation and use. Therefore, we prioritized accessibility over complexity. For example, we ensured that data visualizations were simple and reflected data use for decision-making best practices that allowed for broad understanding from community to national levels. Additionally, given the program's multiple behavior change objectives, we selected a priority set of core indicators to track most frequently over time. The selected indicators were those that we considered most critical to achieving the desired changes or that could be considered a proxy for success or progress against other indicators. For example, 1 core indicator monitored was antenatal care attendance with the assumption that it would be a gateway behavior for other desired behavior changes, such as uptake during antenatal care visits of intermittent preventive treatment of malaria during pregnancy (Figure 2).

## SBC ADAPTIVE MANAGEMENT FRAMEWORK CONTRIBUTIONS TO PROGRAM RESULTS

Application of the SBC Adaptive Management Framework was an important factor in USAID Tulonge Afya's success, contributing to improvements in the scale, saturation, and quality of activities. Given the complex nature of the program, we were able to identify and describe how adaptive shifts in program strategies led to desired impacts in ways that would not have been possible with only process data and baseline, midline, and endline data. We describe illustrative examples of these successes.

### Improved Targeting of HIV Programming Led to Increased HIV Testing Yield

**FIGURE 3:**
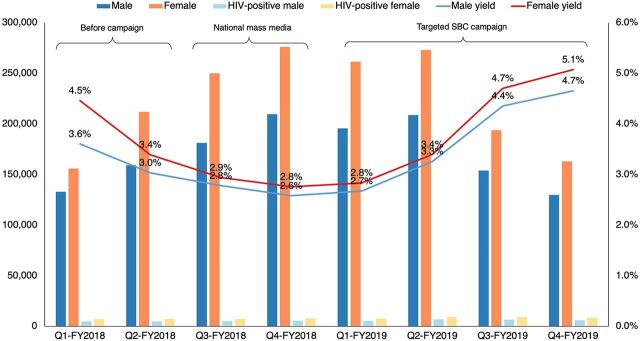
HIV Testing and Positivity Yields by Quarter Within 29 USAID Tulonge Afya–Supported Districts^a^ Abbreviations: FY, fiscal year; SBC, social and behavior change; Q, quarter; USAID, U.S. Agency for International Development. ^a^Chart shows trend of HIV positivity yield by quarter within 29 USAID Tulonge Afya–supported districts, with a dip in positivity yield during the national testing campaign (FY2018 Q3–Q4), followed by a positive rebound and increase in positivity yield as the campaign shifted to more targeted approaches (FY2019 Q1 onward).

In 2018, USAID Tulonge Afya was requested to provide SBC support through its Furaha Yangu campaign to generate awareness of the rollout of Test and Treat—immediate availability of ART upon testing positive for HIV—in Tanzania. The project codesigned and initiated a campaign that promoted awareness of Test and Treat and addressed HIV-related stigma through mass media and other supportive channels, including community-level programming. Although the program was effective in achieving its awareness-raising goals, service delivery data showed declines in HIV testing yield, a priority indicator for both the Government of Tanzania and the U.S. President's Emergency Plan for AIDS Relief, with the increased testing that the campaign generated. The project convened a campaign review meeting with USAID, the Government of Tanzania, and service delivery partners to consider service delivery and campaign data and to identify and gain consensus on recommended changes to the SBC campaign's strategic approach. This resulted in a shift to more targeted SBC activities designed to reach priority populations at higher risk of HIV. Continued monitoring of service data after implementation of these adaptations showed a rebound in HIV testing positivity yield, steadily increasing to levels greater than before the campaign, suggesting USAID Tulonge Afya was effective in reaching individuals at higher risk for HIV with its activities (Figure 3).

The USAID Tulonge Afya project met with stakeholders to review service delivery and campaign data and identify recommended changes to the SBC campaign's strategic approach that could increase HIV testing yield.

### More Efficient Allocation of Resources Improved Reach Among Young Parents and Caregivers

Community-level SBC activities supported by USAID Tulonge Afya were delivered by local CSO partners and their community volunteers. Peer champions led youth-focused activities under the Sitetereki platform. Community volunteers led activities under the Naweza platform and Furaha Yangu campaign. Because of the larger array of activities for which the community volunteers were responsible compared to peer champions, in many districts, reach targets for Naweza and Furaha Yangu were not met compared to reach targets for activities under the Sitetereki platform. This issue was identified during monthly review meetings and raised for remediation during check-ins with community partners and as part of project quarterly data reviews (Step 3 of the framework). A proposed action to address this performance imbalance was to train a select group of older peer champions to conduct Naweza activities with new parents, pivoting from their role in supporting Sitetereki activities. This solution was discussed with CSOs during routine check-in and review meetings, and they found this to be appropriate and feasible. The CSOs then worked with the project to provide supplementary training to a subset of peer champions to deliver Naweza activities (Step 4), which was followed by close monitoring of implementation through targeted supportive supervision visits (Step 5). This reallocation of resources contributed to greater reach of Naweza activities among new parents—improving the achievement of targets for this package—without impacting activity quality. For example, in the Geita Region, this shift contributed to increases in achievement of quarterly reach targets from 16% in quarter 2, to 49% in quarter 3, and 88% in quarter 4.

### Greater Engagement of Stakeholders Enhanced Data for Decision-Making Capacities

Through application of the SBC Adaptive Management Framework, the project engaged stakeholders proactively in gathering data, providing feedback on project strategy and activities, reviewing and analyzing data, and informing adaptations. By engaging stakeholders as active participants in this process, they gained hands-on experience in participatory data collection methods, data analysis and interpretation, and QA of SBC activities. For example, health promotion officers and regional and council health management teams participated in project assessments, data reviews, and CSO check-ins and led supportive supervision activities, thus, strengthening skills in data use, QA/QI, adaptive management, and coordination. One district health promotion coordinator shared that the simple and easy-to-use supportive supervision tool enabled the project to make corrective measures and provide real-time feedback to other project staff.

### Limitation Under USAID Tulonge Afya

A limitation of the approach used under USAID Tulonge Afya was that the framework was not used systematically as a capacity-strengthening tool, as it was developed initially to meet the needs of the project. We believe that engagement of a diverse set of stakeholders around key components of the framework at critical points in time, as described earlier, enhanced the capacities of local stakeholders and will result in the tools and systems being sustained beyond the life of the project. However, we recognize this as an area where future application of the framework should be strengthened.

## LESSONS LEARNED

Many health and development projects plan to apply an adaptive management approach and/or collaborating, learning, and adapting practices. However, these approaches often end up siloed as an M&E responsibility or are deprioritized once implementation has begun. In large integrated SBC projects, these approaches are challenged by the complexities of strategies seeking to shift a range of behaviors and determinant-related indicators. This results in an overemphasis on using data to address process- or output-related indicators rather than intermediary and outcome-related data tied to an SBC theory of change. Our experience applying an SBC Adaptive Management Framework serves as an important case study to better understand how the use of an adaptive management approach, like RF, can be a key contributor to success in complex SBC programs and the critical conditions necessary for the effective establishment and use of such an approach.

### Stakeholder Buy-In and Commitment Is Required To Allocate Adequate Time and Resources

At the outset, an adaptive management approach requires buy-in from the project leadership, staff, donor, and stakeholders. Allocation of time and resources to develop the tools and systems outlined in the framework was essential to lay the foundation for an effective adaptive management approach. This buy-in and commitment must continue through the project life cycle to ensure that ongoing adaptive management activities are prioritized and resourced adequately. We recommend that: (1) SBC programs plan for using a systematic adaptive management approach as part of their program monitoring and evaluation and implementation plans; (2) SBC programs set aside funds not only for data collection and analysis but also for other activities required to implement an adaptive management approach, including tool and system development (e.g., dashboards and mobile applications), and staff and stakeholder time for participation in data review and decision-making activities; and (3) when reviewing proposals, workplans, and budgets, donors advocate for programs to include funds and resources for adaptive management systems and activities. We acknowledge that costing data related to implementation of adaptive management approaches are currently limited and that the level of resources required to effectively implement an adaptive management approach is likely to vary depending on country context, project size and scope, and availability of existing data and tools. We note that it would be valuable for future programs to collect and share cost data that can support better-informed budgeting and planning for use of adaptive management approaches.

Buy-in and commitment from all stakeholders must continue through the project life cycle to ensure that ongoing adaptive management activities are prioritized and resourced adequately.

### Effective Implementation Requires Strong Stakeholder Relationships and Partnerships

Effective implementation of an adaptive management approach also requires programs to have strong relationships with government and donor counterparts to ensure they understand and support the approach. Without these relationships, it can be challenging to communicate the need for adaptive actions or changes in strategy and to obtain support or consensus around these. The shift in the Furaha Yangu campaign approach described earlier serves as a good example of this. Initially, the Government of Tanzania wanted to continue using campaign channels that would achieve widespread reach and broad awareness of Test and Treat. Through collaborative, data-driven meetings, we were able to reach consensus on campaign adaptations that would ensure we were increasing uptake of HIV testing and continuing to promote awareness of Test and Treat, with a focus on population segments at highest risk of HIV and for whom these services were most relevant. In this and other cases, USAID Tulonge Afya conducted ongoing advocacy with USAID and government counterparts to show how use of the framework and resultant programming shifts were enhancing progress toward stated project objectives rather than being seen as setbacks or failures. For this advocacy, the dashboards and data visualizations the project used for decision-making were some of our most effective assets, clearly demonstrating where successful approaches were ready for scale and where there were gaps in need of a change in strategy. We recommend SBC programs engage key stakeholders proactively around the framework as part of program inception to gain support for the approach and to set shared expectations for how application of the framework will inform program strategy and activities, including the potential for program shifts during implementation. Future use of the framework by FHI 360 in SBC projects in Ghana and Ethiopia will introduce this adaptive management approach as a core program element during start-up to generate support for its use and ensure that framework tools, approaches, and principles are embedded within the project life cycle and recognized as core values for all team members and stakeholders from the outset.

We recommend SBC programs engage key stakeholders proactively around the framework as part of program inception to gain support for the approach and set shared expectations.

### Adaptive Management Should Be Embedded Within Project Implementation

An adaptive management approach must be embedded within a project's routine implementation cycle rather than being viewed as an “add-on.” Adaptive management activities should be incorporated within annual workplans and included in annual budgets. For USAID Tulonge Afya, it was important that this also flowed down to partners and subgrantees. Further, participation in adaptive management activities should be an expectation of all staff. While the level of engagement in these activities may differ depending on team members' roles, we recommend that programs include expectations around data use and learning in all project staff job descriptions. Donors may further reinforce this by including adaptive management competencies as desired skills for project key personnel.

### Stakeholders Should Collaborate and Coordinate Activities to Optimize Effort, Time, and Resources

As discussed, a key element of the SBC Adaptive Management Framework is engagement of a diverse array of stakeholders. However, at times, USAID Tulonge Afya faced challenges in obtaining stakeholder participation in framework activities as a result of competing priorities. Though some donors have attempted to encourage use of a common adaptive management approach across programs, there is often little coordination between implementing partners in the use of these approaches. For example, data collection and data review activities are often project-specific, as opposed to identifying opportunities for collaborative activities at the district or regional level. This can contribute to duplicative data collection, competition for time and resources, data overload, and poorly coordinated adaptive actions. As donors continue to require frequent and increasingly granular data reporting and more nimble, adaptive programming from implementers, this challenge is likely to continue. To counteract this, donors should encourage coordinated adaptive management activities across projects working in common areas and, wherever possible, collaborative data collection and analysis activities that minimize duplication of effort. In addition, we note that the first stage of the RF approach, in which stakeholders are convened and an engagement plan with roles and responsibilities is developed, would be of value in overcoming this challenge.

### Promote a Culture of Sharing Failures to Encourage Learning and Adaptation

It is crucial for projects and donors to establish and foster a culture of testing and learning to effectively apply an adaptive management approach. For USAID Tulonge Afya, we found that it was important for our team to feel comfortable acknowledging failures, be willing to have our assumptions challenged, and be flexible and open to new ideas—even if they came from outside the team. A critical element of this was having the support of an agreement officer's representative—the technical expert from USAID who leads the management and monitoring of grants and cooperative agreements. Applying a truly adaptive approach meant accepting that we may not have gotten things right the first time and that we needed to routinely revisit strategies and plans to align them with our latest learning. Initially, it can be challenging to admit when assumptions do not hold. The results and target-based approach common to development programming discourages discussion of failures or acknowledgment of the need for changes in strategy. While USAID Tulonge Afya experienced this, a culture of testing and learning began to build when we saw early programmatic shifts yielding positive results. Additionally, the emergence of COVID-19 in 2020, at the midpoint of the project, necessitated major changes in approach—for example, adaptations to the format of community-based activities and increases in the use of virtual platforms for meeting and collaboration—that encouraged the project to be open to new ideas and ways of thinking that may not have emerged otherwise. We recommend that donors and influential stakeholders support opportunities to encourage more openness and sharing among implementers, such as “fail fests” or after-action reviews to discuss activities and approaches that did not yield anticipated results and inclusion of learning questions within project plans that specifically aim to identify programmatic gaps and opportunities to improve.

Applying a truly adaptive approach meant accepting that we may not get things right the first time and that we needed to routinely revisit strategies and plans.

We recommend that donors and influential stakeholders support opportunities to encourage more openness and sharing among implementers.

The culture of testing and learning was also fostered by project leadership through their active participation in adaptive management activities and emphasis on data-informed decision-making. We recommend that programs provide a variety of regular opportunities for project staff and stakeholders to provide feedback and make recommendations. Annual pause and reflect sessions were of value to the project. However, often these events are conducted at a high level, and project staff, other stakeholders, and community members did not always feel comfortable voicing their opinions in such settings. Monthly CSO check-ins, gathering of participant feedback at the end of activities, and regular internal data reviews provided “lower-risk” settings in which a wider array of voices and perspectives could be reflected, supporting greater inclusion in the project.

### Prioritize Indicators and Develop Tools Guided by a Theory of Change

The SBC Adaptive Management Framework closely aligns with RF on the importance of a theory of change to guide adaptive management activities. This can protect against the inclination to collect as much data as possible without a clear rationale or plan for how it will be used. For USAID Tulonge Afya, we consistently referred to the behavior change and determinant-related intermediary objectives outlined in our SBC strategies to ensure that data being collected always linked back to these objectives. Prioritizing a smaller number of focal indicators that were monitored more routinely also allowed us to be more efficient and focused in both our data collection and use. We also found that the tools we used to drive our adaptive management approach did not need to be complex to be useful. USAID Tulonge Afya emphasized platforms that were widely accessible (i.e., Microsoft Excel, Word, and Power BI) to create simple checklists, trackers, and visualizations. In doing so, we ensured that these tools were accessible for use and adaptation by local government and partners beyond the life of the project. We recommend that SBC programs: (1) use evidence to develop a theory of change for the project; (2) use the project's theory of change to directly inform the selection of indicators that are tracked over time; (3) prioritize a core set of priority or sentinel indicators for routine tracking; and (4) select or develop adaptive management tools that are accessible and meet the skill level of the entire project team, not only the M&E staff.

### Projects Need to Be Flexible and Responsive in Applying Adaptive Management Approaches

Finally, the way in which projects apply adaptive management approaches also needs to be flexible and responsive. While there are core components and principles that are consistent across adaptive management approaches, implementation of these approaches should be tailored to the program and context. It is also important to reflect on what is and is not working within the approach, including the data that are collected and used, and make changes accordingly. This might include changes to which stakeholders are involved at certain decision points or how routinely project plans are reviewed and revised. For example, USAID Tulonge Afya initially intended to update the project's SBC strategies annually. However, we found that this did not allow sufficient time for the effects of high-level strategy changes to become apparent. Therefore, we focused on reviewing and revising activity packages and implementation plans on an annual basis, with strategy review taking place in response to the results of the midterm assessment and as needed based on policy or funding changes.

## IMPLICATIONS

Our experience applying the SBC Adaptive Management Framework within the USAID Tulonge Afya project demonstrates that integration of a systematic adaptive management approach can lead to greater scale, saturation, and quality execution of activities, contributing to measurable improvements in key behaviors and their determinants. It was critical in enabling the project to better understand and program according to our theory of change, thus guiding our use and application of data to routinely adapt and refine our strategies and approaches. For a complex, integrated program, the framework was essential in helping us to focus our MERL activities and direct how to use data and information effectively to plan, implement, and track change over time and across a range of indicators.

The framework was essential in helping us to focus our MERL activities and direct how to use data and information effectively to plan, implement, and track change over time and across a range of indicators.

Successful application of the framework necessitated sufficient investment of time and resources to establish tools and systems and ensure integration and prioritization of adaptive management activities throughout the life of the project. Donors and project implementers need to take these resource requirements into consideration when designing and budgeting for programs. We also found that fostering a strong culture of testing and learning required the buy-in and participation of all members of the project team, as well as external stakeholders and decision-makers. In many cases, this may require individual and organizational behavior change. Leadership from the senior project staff, donor, and decision-makers is critical in setting the tone for this culture. They must prioritize active participation in adaptive management activities; demonstrate openness to new ideas, flexibility, and data-driven decision-making; and recognize that checking assumptions and acknowledging the need for different approaches does not represent project failure but rather facilitates project growth. Donors also have an important role in coordinating the adaptive management activities of implementing partners and encouraging collaboration around shared frameworks and approaches. Opportunities to share feedback and suggestions routinely in a variety of settings can also be beneficial in elevating the voice of communities and project participants, especially in cases where they may not be engaged or feel empowered to participate in larger project reviews, such as pause and reflect sessions. For projects with an online presence, they may also consider using social listening approaches to better understand the perspectives of their target communities related to project implementation. One of the key strengths of USAID Tulonge Afya's approach was the accessibility of the tools and activities used to facilitate the adaptive management approach. In large part, they did not require specialized technology or advanced M&E skills. This can be helpful in maximizing participation, as well as supporting the sustainability of tools and systems in the longer term.

## CONCLUSION

Many development programs seek to apply approaches that incorporate routine collection and use of data and feedback to inform programmatic shifts, with varying levels of success. Under the USAID Tulonge Afya project in Tanzania, we applied the SBC Adaptive Management Framework, which shares many highlighted commonalities with the RF approach, including routine, purposeful inclusion of an array of stakeholders in adaptive management activities; close collaboration between M&E and programmatic staff to ensure data collection and use is informed by, and contributes to ongoing refinement of, the project's theory of change; and regular opportunities for learning and course correction embedded within the project's life cycle. Application of this framework led to measurable improvements in project implementation and achievement of results, driven by an improved understanding of project progress and more purposeful application of data in informing the project's next steps. Learning from the USAID Tulonge Afya experience can contribute to an improved understanding of the conditions necessary to facilitate effective integration of an adaptive management approach within complex SBC programs and the benefits of such an approach for improved implementation and impact.

## Notes

**Peer Reviewed**

**First published online:** May 8, 2023.
